# Health-Promoting Lifestyle and Psychosocial Correlates in Patients with NAFLD/MASLD and Family Members: A Comparative Cross-Sectional Study of Independent Samples

**DOI:** 10.3390/healthcare14172686

**Published:** 2026-08-24

**Authors:** Yuhuan Yu, Lili Ji

**Affiliations:** 1 Department of Gastroenterology (Ward II), Affiliated Hospital of Shandong Second Medical University, Weifang 261031, China; 2 School of Nursing, Shandong Second Medical University, Weifang 261053, China

**Keywords:** self-efficacy, perceived social support, autonomous motivation, electronic questionnaire, convenience sampling, health behavior

## Abstract

**Background/Objectives:** Lifestyle modification is central to non-alcoholic fatty liver disease, now termed metabolic dysfunction-associated steatotic liver disease (NAFLD/MASLD), yet comparable health-promoting lifestyle (HPL) data for patients and family members are limited. This study compared two independent samples and examined psychosocial correlates. **Methods:** Two convenience samples recruited at one tertiary hospital were analyzed: 305 adults with NAFLD/MASLD and 299 adult family members of affected patients. A self-administered electronic questionnaire comprised the Health-Promoting Lifestyle Profile II (HPLP-II), the Self-Rated Abilities for Health Practices scale, autonomous-motivation items from the Treatment Self-Regulation Questionnaire, the Medical Outcomes Study Social Support Survey, and sociodemographic items. Group comparisons used parametric and non-parametric tests. HPL correlates were examined using complete-case regression, HC3 inference, and rank-based sensitivity analysis. Cross-sectional indirect-association decompositions used 5000 bootstrap resamples. **Results:** HPLP-II scores were 102.2 ± 17.5 in patients and 104.2 ± 17.4 in family members (*p* = 0.171; Mann–Whitney *p* = 0.166); the adjusted patient-minus-family difference was −1.14 (HC3 95% CI −3.89 to 1.60; *p* = 0.413). Physical activity was the lowest-scoring dimension. Self-efficacy was the most robust correlate in both samples; social support was associated in the primary models but weakened under rank transformation in family members. Group-by-correlate interactions were not significant. Exploratory indirect associations through self-efficacy were 0.355 (95% bootstrap CI 0.234–0.512) in patients and 0.163 (0.071–0.287) in family members; reverse-order models were also non-zero. **Conclusions:** The independent unmatched samples reported similar moderate, physically inactive lifestyles. The results identify associations, not shared-household effects or causal mechanisms; objectively characterized, dyad-matched longitudinal studies are needed.

## 1. Introduction

Non-alcoholic fatty liver disease (NAFLD), renamed metabolic dysfunction-associated steatotic liver disease (MASLD) in 2023, is the most common chronic liver disease worldwide and has increased rapidly in China [1,2,3,4]. Current international and Chinese guidance places sustained lifestyle modification at the center of management [5,6]. Dietary change, increased physical activity, and gradual weight reduction can improve steatosis and histological features [7,8], while favorable lifestyle patterns are associated with lower mortality among people with NAFLD [9]. Recent evidence syntheses emphasize structured, personalized, multidisciplinary support rather than one-time advice [10]. We therefore use NAFLD/MASLD throughout, retaining NAFLD where it describes the diagnosis used during recruitment or the terminology of earlier studies.

A health-promoting lifestyle (HPL) is a multidimensional pattern of self-initiated actions covering nutrition, physical activity, health responsibility, stress management, interpersonal relations, and spiritual growth [11,12]. Research specifically examining self-management in NAFLD remains limited. A Korean cross-sectional study found moderate self-management and identified self-efficacy as an important correlate [13]. Qualitative work in China additionally identified planning, coping, feedback, action control, and self-efficacy as facilitators of adherence to lifestyle prescriptions [14].

Family context is relevant to chronic-disease behavior, but it must not be inferred without an appropriate dyadic design. Hepatic steatosis and fibrosis show familial aggregation [15], and family behavior and communication can influence chronic-illness outcomes [16,17]. However, the present patient and family-member records were not reliably linkable as dyads. Accordingly, this study compares two independent clinical samples and does not estimate within-household concordance, partner effects, or household clustering.

Social cognitive theory identifies self-efficacy as a modifiable influence on health behavior [18,19], operationalized here using the Self-Rated Abilities for Health Practices scale [20]. Self-determination theory distinguishes autonomous from controlled motivation [21,22], although autonomous motivation does not guarantee action when opportunity, skills, or disease-related constraints are limiting [23]. Perceived social support may facilitate coping and behavior change [24,25] and was assessed with the Medical Outcomes Study Social Support Survey [26].

This study addressed three research questions: (1) Do HPL level and profile differ between the patient and family-member samples; (2) Are self-efficacy, perceived social support, and autonomous motivation associated with HPL in each sample, and do their estimates differ by group; (3) Is part of the cross-sectional association between perceived social support and HPL statistically shared with self-efficacy? Family-context evidence motivated the comparison but, because the records were not linkable dyads, no household-concordance hypothesis was tested [15,16,17]. Social cognitive theory and prior NAFLD self-management evidence supported the expectation that self-efficacy would be positively associated with HPL [13,18,19,20]. Evidence on social support supported a positive association, whereas self-determination theory and implementation constraints suggested that autonomous motivation might have a weaker unique association after simultaneous adjustment [21,22,23,24,25,26]. Accordingly, the following analytical expectations guided the complete re-analysis: H1, the adjusted difference in HPLP-II total score would be small; H2, self-efficacy and perceived social support would be positive correlates of HPL in both samples; and H3, a positive indirect statistical association through self-efficacy would be observed. These expectations were not prospectively registered and are not presented as preregistered confirmatory hypotheses. Reporting follows STROBE [27]. Because all main constructs were self-reported in one administration, common-method bias was considered explicitly [28]. Cross-sectional indirect-association models cannot establish temporal mediation [29,30,31].

## 2. Materials and Methods

### 2.1. Study Design and Setting

This single-center comparative cross-sectional study was conducted at a tertiary teaching hospital in Weifang, Shandong Province, China. Platform timestamps for completed questionnaires ranged from 11 July 2025 to 15 June 2026. Both samples were analyzed as independent groups.

### 2.2. Participants, Recruitment, and Sample-Size Rationale

Two convenience samples were recruited. Eligible patients were adults aged at least 18 years with an established clinical and ultrasonographic diagnosis of NAFLD under the diagnostic framework used at the time of recruitment, able to read and complete the questionnaire, and willing to provide written informed consent. Exclusion criteria were substantial alcohol consumption or another identifiable cause of hepatic steatosis, decompensated cirrhosis, hepatocellular carcinoma, or a cognitive or psychiatric condition precluding participation. The comparison sample comprised adult family members of patients with NAFLD/MASLD who could consent and reported no previous diagnosis of fatty liver disease. The retained files do not establish that every family participant cohabited with a corresponding enrolled patient.

Study personnel explained the study and stated eligibility criteria, obtained written consent, and then provided a link to the electronic questionnaire. The archived exports contain completed records only and no auditable screening or recruitment log. Consequently, the numbers screened, invited, declining, or excluded before export—and therefore response rates—cannot be reconstructed. We do not claim consecutive screening, hospital-record verification of every eligibility criterion, recruitment incentives, or a specific number of discarded submissions because supporting source documentation was unavailable.

No reliable shared dyad identifier or relationship-to-enrolled-patient field was retained across the two exports. A patient record therefore cannot be linked confidently to a particular family-member record. Dyadic models, household random effects, and spouse-only analyses were not possible, and all statistical tests treated groups as independent. Dyadic analyses would require prospectively linked pairs [32].

Family-member NAFLD/MASLD status was based only on self-report of no known fatty liver diagnosis. Objective liver imaging, liver biochemistry, and fibrosis assessment were not collected for this comparison sample. Undiagnosed disease is therefore possible and could bias between-group estimates toward similarity.

The original study planning used a pragmatic heuristic of at least 10–15 observations per candidate regression predictor and aimed for at least 150 participants per group; no formal prospective effect-size calculation or preregistration was documented. With six predictors in the primary model, the final complete-case samples of 296 and 290 exceeded common regression heuristics [33]. As a retrospective sensitivity description, the full group sizes could detect a standardized independent-group difference of approximately d = 0.23 at 80% power (two-sided α = 0.05); this calculation does not validate the design retrospectively or exclude smaller effects.

### 2.3. Measures

Sociodemographic and clinical variables. The structured form captured age, sex, education, marital status, medical insurance, living arrangement, sleep duration, smoking and alcohol use, and self-reported height and weight. Body mass index (BMI) was calculated as kg/m^2^ and categorized using Chinese cut points: <24.0, 24.0–27.9, and ≥28.0 kg/m^2^. Height and weight were not measured by investigators or standardized equipment; self-report can bias BMI [34]. Disease duration was derivable for a subset of patients. Alanine and aspartate aminotransferases, lipid profile, imaging severity, fibrosis stage, and comorbidity indices were unavailable.

Health-promoting lifestyle: The Chinese HPLP-II contained 40 items across interpersonal relations, nutrition, health responsibility, stress management, physical activity, and spiritual growth. Items were scored from 1 (never) to 4 (routinely); total scores ranged from 40 to 160, with higher scores indicating healthier behavior [11,12]. The physical-activity dimension included planned exercise, vigorous activity, light-to-moderate activity, recreational activity, stretching, activity incorporated into daily life, pulse monitoring, and exercising within a target heart-rate range. It therefore covered structured and everyday activity but did not measure occupational physical activity as a separate domain.

Self-efficacy for health practices: The 28-item Self-Rated Abilities for Health Practices scale assessed nutrition, psychological well-being, exercise, and health responsibility. Items were scored from 0 (no confidence) to 4 (complete confidence), producing a total range of 0–112 [20].

Autonomous motivation: Twelve autonomous-regulation items from the Treatment Self-Regulation Questionnaire were scored from 1 (strongly disagree) to 5 (strongly agree), yielding a total range of 12–60 [21,22,23].

Perceived social support: The 19-item Medical Outcomes Study Social Support Survey assessed emotional/informational, tangible, affectionate, and positive social-interaction support. Items were scored from 1 to 5; total scores ranged from 19 to 95 [24,25,26].

### 2.4. Electronic Data, Cleaning, and Quality Control

The self-administered questionnaire was distributed through an online survey platform via WeChat. Scale items were completed in every exported record; demographic fields were cleaned after export. Dates of birth were parsed conservatively against submission timestamps, and ages outside 18–90 years were treated as missing. Height values between 1 and 3 were interpreted as meters and converted to centimeters; other values were treated as centimeters. Calculated BMI values < 15 or >60 kg/m^2^ were treated as implausible and set to missing. No outcome or scale value was imputed. The retained platform exports did not include IP addresses, stable device identifiers, or an exclusion audit sufficient to verify whether an IP- or device-level duplicate-prevention rule had been applied. Duplicate-control settings therefore could not be confirmed retrospectively, and no unverified prevention claim is made.

### 2.5. Ethical Considerations

The study was conducted in accordance with the Declaration of Helsinki and was approved by the Ethics Committee of the Affiliated Hospital of Shandong Second Medical University on 30 April 2025 (approval no. SDSMU-2025-ky-185). Written informed consent was obtained before questionnaire completion. All platform-recorded questionnaire dates used in this analysis occurred after approval.

### 2.6. Statistical Analysis

The analysis was reproduced locally in Python 3.12.13 using pandas 3.0.1, NumPy 2.3.5, SciPy 1.18.0, statsmodels 0.14.6, and scikit-learn 1.9.0. Continuous variables are summarized as mean ± SD and categorical variables as *n* (%). Independent-samples *t*-tests and chi-square tests were used for descriptive comparisons; Welch tests were used when variance differed. Cohen’s d quantified standardized mean differences. Because Shapiro–Wilk tests and plots showed departures from normality, Mann–Whitney U tests accompanied group comparisons and Spearman coefficients accompanied Pearson correlations.

The adjusted group difference in HPLP-II total score was estimated with ordinary least squares including group, age, sex, and BMI. Primary within-group models regressed HPLP-II total score on self-efficacy, perceived social support, autonomous motivation, age, sex, and BMI, entered simultaneously without stepwise selection. Analyses used complete cases; 3 patient and 6 family-member age values and 6 patient and 3 family-member BMI values were missing, leaving *n* = 296 and *n* = 290. Heteroscedasticity-consistent HC3 confidence intervals and *p* values were the primary inference. Residual-versus-fitted and Q–Q plots, Shapiro–Wilk, Breusch–Pagan, Ramsey RESET, Durbin–Watson, and variance-inflation factors were examined. Rank-transformed regression served as a distribution-robust sensitivity analysis.

A pooled model tested group-by-self-efficacy, group-by-support, and group-by-motivation interactions while adjusting for age, sex, BMI, and main effects. Sex-stratified group comparisons and a group-by-sex interaction were also reported. A non-significant interaction was interpreted as absence of statistical evidence for heterogeneity, not proof of equivalence.

**Figure 1:**
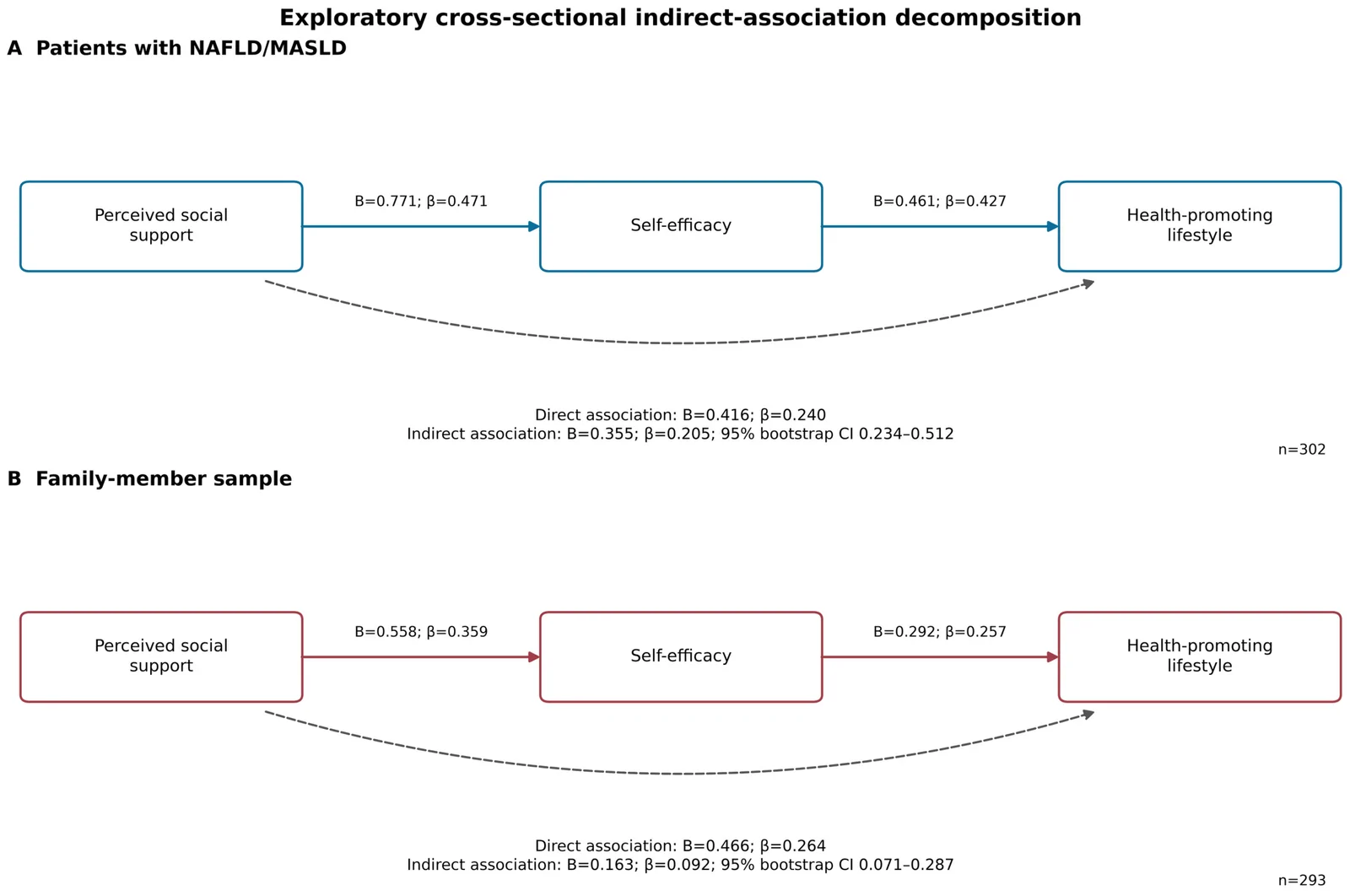
Exploratory regression-based cross-sectional indirect-association decomposition. Age, sex, and BMI are displayed as measured background factors for both samples. The reported regression-decomposition coefficients were adjusted for age and sex; BMI was not included in these specific equations and is shown to make the measured background-factor set explicit. This diagram summarizes ordinary least-squares regression coefficients; it is not a PLS-SEM or structural-equation model. B denotes unstandardized coefficients and β standardized coefficients. The dashed direct association is conditional on self-efficacy. Bootstrap intervals used 5000 bias-corrected resamples. Arrows are statistical notation only and do not indicate temporal or causal direction; reverse-order models were also supported.

The social-support–self-efficacy–HPL sequence was examined as an exploratory regression decomposition adjusted for age and sex, with 5000 bias-corrected bootstrap resamples for the indirect association [29,30,31]. Five thousand resamples were selected a priori as a conventional compromise that reduces Monte Carlo variability and stabilizes percentile estimates while retaining reasonable computational cost; materially smaller pools can yield less stable interval limits, whereas substantially larger pools usually offer diminishing practical gains. Standardized and unstandardized coefficients were reported. Figure 1 additionally displays age, sex, and BMI as measured background factors. The reported coefficients remain from equations adjusted for age and sex; BMI was not included in these specific decomposition equations. A reverse-order model (HPL–self-efficacy–support) was fitted to demonstrate directional ambiguity. The analysis is not evidence of causal mediation.

Internal consistency was summarized using Cronbach’s α. An unrotated single-factor extraction across all scale items was reported only as a limited Harman diagnostic; it cannot exclude common-method bias [28]. Expanded sensitivity models added sleep duration, current smoking, current alcohol use, and—among patients—disease duration. Disease duration was not included in the primary models because it was patient-specific, unavailable for the family-member sample, and derivable for only 81.3% of patients; retaining common covariates in the primary models preserved comparability across groups and avoided additional missing-data loss. Models excluding BMI values >40 kg/m^2^ examined influence from extreme self-reported anthropometry. Two-sided *p* < 0.05 was considered statistically significant; exact *p* values are reported unless *p* < 0.001.

## 3. Results

### 3.1. Participant Characteristics and Completeness

The archived exports contained 604 completed records: 305 patients and 299 family members. The recruitment denominator was unavailable, so no response rate is reported. Scale items were complete. Age was valid for 302 patients and 293 family members; BMI was valid for 299 and 296, respectively. Patients were slightly older, more often male, and had higher BMI and obesity prevalence (Table 1). Because BMI was skewed, medians and interquartile ranges are reported in Table 1: 27.08 kg/m^2^ (25.06–29.54) in patients and 25.90 kg/m^2^ (24.31–27.86) in family members (Mann–Whitney *p* < 0.001). Education, marital status, insurance, and living arrangement did not differ materially between the patient and family-member samples. In a pooled sex-stratified cross-tabulation, education differed by sex (χ^2^(3) = 10.28, *p* = 0.016), driven mainly by the patient sample (χ^2^(3) = 37.56, *p* < 0.001); no sex difference was detected among family members (χ^2^(3) = 3.22, *p* = 0.358). Education is only a proxy, and the study did not directly measure health literacy.

### 3.2. Scale Distributions, Reliability, and Group Comparisons

Cronbach’s α values were high: 0.965 and 0.967 for HPLP-II, 0.968 and 0.970 for self-efficacy, 0.965 and 0.963 for social support, and 0.976 and 0.974 for autonomous motivation in patients and family members, respectively. The first unrotated factor accounted for 33.3% and 29.8% of total item variance. These values do not rule out item redundancy or common-method bias. Shapiro–Wilk tests were significant for all continuous study variables, with particularly skewed BMI distributions; histograms and Q–Q plots are provided in Figures S1 and S2. Autonomous-motivation scores showed modest upper-end clustering but not a marked strict ceiling: exact maximum scores occurred in 29/305 patients (9.5%) and 28/299 family members (9.4%); medians were 48 (IQR 38–52) and 48 (41–53), respectively.

Mean HPLP-II total scores were 102.2 ± 17.5 in patients and 104.2 ± 17.4 in family members (t = −1.371, *p* = 0.171, d = −0.112; Mann–Whitney *p* = 0.166; Table 2). The patient-minus-family adjusted difference was −1.14 (HC3 95% CI −3.89 to 1.60; *p* = 0.413; *n* = 586). Physical activity was the lowest HPLP-II dimension in both samples. Family members had a higher interpersonal-relations mean (*p* = 0.007; Mann–Whitney *p* = 0.009). The self-efficacy difference was nominally significant by *t*-test (*p* = 0.047) but not by Mann–Whitney test (*p* = 0.082).

Among women, the parametric patient–family comparison was *p* = 0.040 (Welch *p* = 0.038; d = −0.237), but the Mann–Whitney result was *p* = 0.059. Among men, *p* = 0.543 (Mann–Whitney *p* = 0.722). The adjusted group-by-sex interaction was not significant (B = −5.01; HC3 *p* = 0.081). Thus, the estimates differed by sex, but the data do not establish interaction or equivalence.

### 3.3. Correlations and Primary Regression Models

HPLP-II total scores correlated positively with all three psychosocial measures by Pearson analysis (Table 3). Spearman analyses confirmed self-efficacy and social-support associations in both samples. The autonomous-motivation Spearman association was significant in patients but not in family members (ρ = 0.106, *p* = 0.068).

The patient primary model included 296 complete cases (R^2^ = 0.353; adjusted R^2^ = 0.340). Self-efficacy (B = 0.498, β = 0.463; HC3 *p* < 0.001) and perceived social support (B = 0.450, β = 0.259; HC3 *p* = 0.001) were associated with HPLP-II total score (Table 4). The family-member model included 290 cases (R^2^ = 0.290; adjusted R^2^ = 0.275); self-efficacy (B = 0.359, β = 0.314; HC3 *p* < 0.001), social support (B = 0.510, β = 0.287; HC3 *p* < 0.001), and younger age (B = −0.305; HC3 *p* = 0.002) were associated with HPL. Family BMI was significant with conventional OLS but not HC3 inference (HC3 *p* = 0.145) and was unstable in sensitivity analyses, so it is not interpreted as an independent correlate.

All variance-inflation factors were ≤1.56. The patient model showed no Breusch–Pagan or RESET evidence of major misspecification, although residual normality was rejected. The family model showed heteroscedasticity and RESET evidence of nonlinearity; HC3 inference and rank-based models were therefore emphasized. In rank-based models, self-efficacy remained associated in both samples and social support remained associated in patients, whereas the family social-support estimate weakened (*p* = 0.128). Full diagnostics are in Table S1 and Figure S3.

None of the pooled group-by-correlate interactions was significant using HC3 inference: self-efficacy B = −0.142 (95% CI −0.361 to 0.078; *p* = 0.205), social support B = 0.086 (−0.274 to 0.446; *p* = 0.638), and autonomous motivation B = −0.156 (−0.560 to 0.248; *p* = 0.448). These results indicate no detected heterogeneity; they do not prove identical effects.

### 3.4. Sensitivity Analyses

Expanded models retained 279 patients and 289 family members (Table 5). Self-efficacy and social support remained associated with HPL in both; sleep duration, smoking, and alcohol use were not significant. Patient disease duration was positively associated (B = 0.755, *p* = 0.010), but clinical severity measures were unavailable. Excluding BMI > 40 kg/m^2^ did not materially change the self-efficacy or support estimates. Family BMI became more negative after this exclusion, reinforcing its instability rather than a robust linear interpretation. Nine records per group were excluded from the primary model because of age or BMI missingness; included and excluded records did not differ significantly on HPLP-II or the three psychosocial scores (all Mann–Whitney *p* > 0.26; Table S4).

### 3.5. Exploratory Indirect-Association Decomposition

Among patients (*n* = 302), the unstandardized indirect association of social support with HPL through self-efficacy was B = 0.355 (standardized 0.205; 95% bias-corrected bootstrap CI 0.234–0.512); the direct association was B = 0.416 (standardized 0.240). Among family members (*n* = 293), the indirect association was B = 0.163 (standardized 0.092; 95% CI 0.071–0.287) and the direct association was B = 0.466 (standardized 0.264; Figure 1). Reverse-order HPL–self-efficacy–support models also produced non-zero indirect associations: B = 0.104 (95% CI 0.057–0.162) in patients and B = 0.055 (0.018–0.101) in family members (Table S3). This directional ambiguity is expected in contemporaneous data and precludes a causal or temporal interpretation.

## 4. Discussion

This re-analysis provides three main findings. First, HPLP-II totals were similar in the two independent samples after demographic adjustment and under non-parametric analysis, while physical activity was the lowest-scoring dimension in both. Second, self-efficacy was the most consistent psychosocial correlate of HPL. Perceived social support was associated in the primary HC3 models, but the family-sample estimate was less stable under rank transformation. Third, the social-support–self-efficacy–HPL decomposition produced non-zero indirect associations, but reverse-order models did as well; the result is descriptive rather than mechanistic.

Viewed against the prespecified research questions, RQ1/H1 was supported descriptively: the adjusted group difference was small and non-significant, although the independent-sample design prevents a household interpretation. For RQ2/H2, self-efficacy was the most consistent positive correlate, and perceived social support was positive in the primary models, while autonomous motivation did not retain an independent association; the non-significant group interactions indicate no detected heterogeneity rather than equivalence. For RQ3/H3, the positive indirect statistical association through self-efficacy was observed, but the reverse-order result and contemporaneous measurement prevent a temporal or causal interpretation. These findings are consistent with prior NAFLD self-management evidence and the theoretical roles of efficacy, support, and implementation constraints [13,14,18,19,20,21,22,23,24,25,26].

The moderate HPL level and prominence of physical inactivity are consistent with prior NAFLD self-management research [13,14]. The small adjusted group difference should not be interpreted as household concordance: the records were not linked dyads, and some family members may have had undiagnosed NAFLD/MASLD. Misclassification and selection through a single-center electronic convenience sample could both make groups appear more similar. The nominal women-only *t*-test difference, its weaker rank-test result, and the non-significant group-by-sex interaction also show why the overall null comparison cannot be attributed confidently to sex composition.

The self-efficacy findings align with social cognitive theory and with evidence that changing self-efficacy is linked to health intentions and behavior [18,19,35]. Self-efficacy remained associated after adding available behavioral covariates and in rank-based models. Social support may provide encouragement, information, and practical help, yet its family-sample estimate was sensitive to the model scale; future longitudinal research should test when support is enabling, neutral, or controlling.

Autonomous motivation correlated weakly with HPL and was not an independent correlate after the other psychosocial measures were entered. This does not refute self-determination theory. Autonomous motivation may share variance with self-efficacy, and motivated intentions may not translate into action without planning, capability, time, safe exercise options, or feedback [14,21,22,23]. Restricted score variation and the cross-sectional measurement context may also attenuate its unique coefficient.

The results support candidate components for future trials, not an immediate clinical recommendation. A graded physical-activity program could combine individualized goals, self-monitoring, feedback, problem solving, and supportive family communication. Smartphone-assisted MASLD interventions can deliver repeated lifestyle content [36], and family-setting mHealth and chronic-disease trials demonstrate feasible ways to involve relatives and build self-management support [37,38]. However, an app-only approach may exclude older adults or participants with limited digital literacy. Future trials should screen digital access and skills, provide brief hands-on training and large-font/simple interfaces, allow caregiver or clinician assistance, and offer equivalent telephone or paper pathways. Exercise can reduce liver fat in metabolic disease [39]. Whether a hybrid, dyadic, self-efficacy-focused intervention improves objective activity or liver outcomes in NAFLD/MASLD must be tested in a prospectively registered randomized trial.

### Strengths and Limitations

Several limitations materially constrain inference. First, the patient and family-member samples were independent, unmatched convenience samples; no household clustering, actor–partner, spouse-only, or within-family analysis was possible. Second, family-member NAFLD/MASLD status was self-reported without imaging or biochemistry, so undiagnosed disease may have contaminated the comparison group. No robust Weifang-specific adult population estimate was identified to quantify this risk. A meta-analysis did identify a Shandong health-check study in which ultrasonography detected NAFLD in 16.6% of lean/non-obese adults, but that selected sample cannot be treated as an estimate of undiagnosed disease in Weifang or in the present family-member group [40]. Third, the screening denominator, refusal count, pre-export exclusions, incentives, response rate, IP addresses, device identifiers, and duplicate-screening audit were not retained; duplicate prevention cannot be verified, and selection bias cannot be quantified. Fourth, recruitment exclusively from a tertiary gastroenterology ward may overrepresent referred, symptomatic, newly diagnosed, or clinically complex patients and underrepresent mild community-managed disease. Because liver enzymes, imaging severity, and fibrosis stage were unavailable, the direction and magnitude of severity selection cannot be quantified. Fifth, WeChat-based participation may underrepresent older adults, people with low digital literacy, lower socioeconomic resources, or rural residence, limiting generalizability. Sixth, education differed by sex in the pooled sample, especially among patients, and may partly proxy health-literacy or socioeconomic differences; health literacy was not measured directly. Seventh, all main constructs and anthropometry were self-reported in one administration. The HPLP-II covered planned, recreational, and daily-life activity but did not isolate occupational physical activity. Very high α values may indicate item redundancy, and Harman’s diagnostic cannot exclude common-method bias. Eighth, age and BMI cleaning created nine complete-case exclusions per group; although scale scores did not differ detectably between included and excluded records, data may not be missing at random. Ninth, clinical granularity was limited, and coarse sleep, smoking, and alcohol variables cannot eliminate residual confounding. Tenth, non-normality, heteroscedasticity, and family-model nonlinearity required robust and rank-based sensitivity analyses; some estimates, especially family social support and BMI, were not fully stable. Finally, the cross-sectional design precludes temporal or causal claims, and the indirect-association analysis must not be interpreted as mediation.

## 5. Conclusions

Patients with NAFLD/MASLD and a separate sample of family members of affected patients reported similarly moderate, physically inactive health-promoting lifestyles. Self-efficacy was the most robust correlate in both samples; perceived social support was associated in the primary models but was less stable for family members under rank-based sensitivity analysis. The exploratory indirect association through self-efficacy was directionally ambiguous. These findings do not demonstrate shared household behavior or a causal pathway. Objectively characterized, dyad-matched longitudinal studies and randomized trials are required before family-level intervention claims can be made.

## Figures and Tables

**Table 1:** Sociodemographic and anthropometric characteristics.

Characteristic	Patients (*n* = 305)	Family Members (*n* = 299)	Statistic	*p*
Age, years	48.5 ± 11.5 (*n* = 302)	46.5 ± 11.3 (*n* = 293)	t = 2.13	0.034
Sex, *n* (%)			χ^2^ = 22.37	<0.001
Male	177 (58.0)	116 (38.8)		
Female	128 (42.0)	183 (61.2)		
Educational level, *n* (%)			χ^2^ = 1.90	0.593
Primary school	8 (2.6)	6 (2.0)		
Junior secondary	58 (19.0)	54 (18.1)		
Senior secondary	80 (26.2)	93 (31.1)		
College or above	159 (52.1)	146 (48.8)		
Married, *n* (%)	292 (95.7)	285 (95.3)	χ^2^ = 0.06	0.803
Medical insurance, *n* (%)			χ^2^ = 1.36	0.506
Employee	184 (60.3)	167 (55.9)		
Resident	96 (31.5)	107 (35.8)		
Self-paid/public/other	25 (8.2)	25 (8.4)		
Living with family, *n* (%)	295 (96.7)	288 (96.3)	χ^2^ = 0.07	0.788
BMI, kg/m^2^, median (IQR)	27.08 (25.06–29.54) (*n* = 299)	25.90 (24.31–27.86) (*n* = 296)	Mann–Whitney U = 53,883	<0.001
BMI category, *n* (%)			χ^2^ = 18.83	<0.001
<24.0 kg/m^2^	46 (15.4)	65 (22.0)		
24.0–27.9 kg/m^2^	131 (43.8)	159 (53.7)		
≥28.0 kg/m^2^	122 (40.8)	72 (24.3)		

BMI, body mass index; IQR, interquartile range; Percentages use valid denominators. The Mann–Whitney U test was used for the BMI comparison because of skewness. Exact values were rounded; *p* values are two-sided.

**Table 2:** Health-promoting lifestyle and psychosocial scores by group.

Variable	Patients	Family Members	t	*p*	Cohen’s d	MW *p*
HPLP-II total (40–160)	102.2 ± 17.5	104.2 ± 17.4	−1.371	0.171	−0.112	0.166
Interpersonal relations (1–4)	3.11 ± 0.65	3.25 ± 0.57	−2.709	0.007	−0.220	0.009
Nutrition (1–4)	2.68 ± 0.55	2.73 ± 0.57	−1.052	0.293	−0.086	0.296
Health responsibility (1–4)	2.48 ± 0.57	2.54 ± 0.55	−1.291	0.197	−0.105	0.122
Stress management (1–4)	2.65 ± 0.55	2.68 ± 0.55	−0.721	0.471	−0.059	0.398
Physical activity (1–4)	2.16 ± 0.59	2.17 ± 0.59	−0.197	0.844	−0.016	0.943
Spiritual growth (1–4)	2.49 ± 0.54	2.51 ± 0.54	−0.558	0.577	−0.045	0.852
Self-efficacy (0–112)	71.0 ± 16.2	73.5 ± 15.3	−1.991	0.047	−0.162	0.082
Perceived social support (19–95)	67.2 ± 9.9	67.5 ± 9.8	−0.355	0.723	−0.029	0.801
Autonomous motivation (12–60)	46.4 ± 8.2	47.6 ± 8.1	−1.724	0.085	−0.140	0.072

Values are mean ± SD. SD, standard deviation. MW, Mann–Whitney U test. Dimension values are mean item scores. HPLP-II, Health-Promoting Lifestyle Profile II.

**Table 3:** Pearson and Spearman correlations with HPLP-II total score.

Correlate	Patients r (*p*)	Patients ρ (*p*)	Family r (*p*)	Family ρ (*p*)
Self-efficacy	0.539 (<0.001)	0.516 (<0.001)	0.379 (<0.001)	0.230 (<0.001)
Perceived social support	0.447 (<0.001)	0.364 (<0.001)	0.381 (<0.001)	0.205 (<0.001)
Autonomous motivation	0.295 (<0.001)	0.263 (<0.001)	0.154 (0.008)	0.106 (0.068)

**Table 4:** Simultaneous multiple regression of HPLP-II total score.

Predictor	Patients B (HC3 95% CI)	β	HC3 *p*	Family B (HC3 95% CI)	β	HC3 *p*
Self-efficacy	0.498 (0.350 to 0.646)	0.463	<0.001	0.359 (0.199 to 0.519)	0.314	<0.001
Perceived social support	0.450 (0.186 to 0.713)	0.259	<0.001	0.510 (0.260 to 0.760)	0.287	<0.001
Autonomous motivation	−0.129 (−0.390 to 0.131)	−0.062	0.329	−0.238 (−0.569 to 0.092)	−0.111	0.156
Age, years	−0.106 (−0.236 to 0.024)	−0.070	0.109	−0.305 (−0.494 to −0.117)	−0.198	0.002
Male sex	−0.126 (−3.444 to 3.193)	−0.004	0.941	−1.662 (−5.487 to 2.164)	−0.047	0.393
BMI, kg/m^2^	0.080 (−0.163 to 0.323)	0.024	0.516	−0.647 (−1.519 to 0.225)	−0.126	0.145

Patient model *n* = 296, R^2^ = 0.353, adjusted R^2^ = 0.340; family model *n* = 290, R^2^ = 0.290, adjusted R^2^ = 0.275. B is unstandardized; β is standardized. All predictors were entered simultaneously. CI and *p* values use HC3 robust covariance.

**Table 5:** Robustness and sensitivity of the two primary psychosocial estimates.

Analysis	Patients *n*	Patients Estimates (*p*)	Family *n*	Family Estimates (*p*)
Primary HC3 model	296	Self-efficacy 0.498 (<0.001); support 0.450 (0.001)	290	Self-efficacy 0.359 (<0.001); support 0.510 (<0.001)
Expanded covariate model	279	Self-efficacy 0.546 (<0.001); support 0.384 (<0.001)	289	Self-efficacy 0.356 (<0.001); support 0.501 (<0.001)
Exclude BMI > 40 kg/m^2^	290	Self-efficacy 0.485 (<0.001); support 0.456 (<0.001)	287	Self-efficacy 0.316 (<0.001); support 0.431 (<0.001)
Rank-transformed HC3 model	296	Self-efficacy rank B = 0.468 (<0.001); support rank B = 0.188 (0.003)	290	Self-efficacy rank B = 0.194 (0.004); support rank B = 0.100 (0.128)

Expanded models additionally included sleep duration, current smoking, current alcohol use, and patient disease duration. Rank B values are not on the original HPLP-II scale.

## Data Availability

A de-identified analytic dataset and analysis code are available from the corresponding author on reasonable request, subject to ethics and institutional review. Raw questionnaire exports are not publicly available because they contain direct and indirect identifiers and participants did not consent to public deposition.

## Supplementary Materials

The following supporting information can be downloaded at: https://www.mdpi.com/article/10.3390/healthcare14172686/s1, Table S1: Primary regression diagnostics; Table S2: Expanded sensitivity-model coefficients; Table S3: Exploratory indirect-association and reverse-order decompositions; Table S4: Complete-case missingness assessment; Figure S1: Histograms and normal Q–Q plots for continuous study variables in patients; Figure S2: Histograms and normal Q–Q plots for continuous study variables in family members; Figure S3: Residual-versus-fitted and residual normal Q–Q plots for the primary patient and family-member regressions.
