# Supplementary material for: Health-Promoting Lifestyle and Psychosocial Correlates in Patients with NAFLD/MASLD and Family Members: A Comparative Cross-Sectional Study of Independent Samples
Source: Healthcare (Basel). 2026 Aug 24;14(17):2686. doi: 10.3390/healthcare14172686 (PMC13565544; doi:10.3390/healthcare14172686)

Continuous-variable distributions: patients

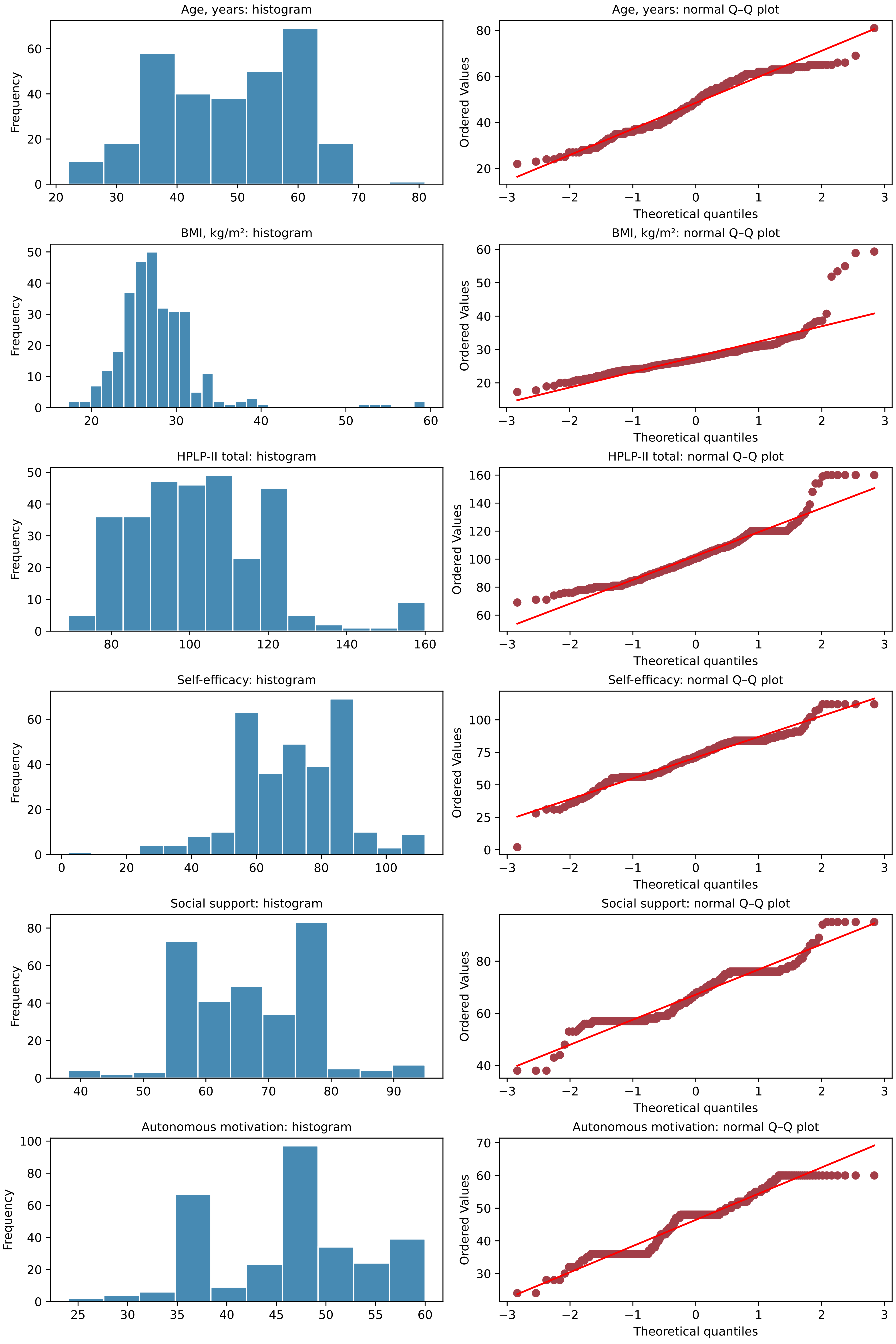

Supplement: Supplementary file 1 [file healthcare-14-02686-s001.zip › Supplementary_Figures/Figure_S1_distributions_patients.pdf]
