# Supplementary material for: Health-Promoting Lifestyle and Psychosocial Correlates in Patients with NAFLD/MASLD and Family Members: A Comparative Cross-Sectional Study of Independent Samples
Source: Healthcare (Basel). 2026 Aug 24;14(17):2686. doi: 10.3390/healthcare14172686 (PMC13565544; doi:10.3390/healthcare14172686)
Supplement: Supplementary file 1 [file healthcare-14-02686-s001.zip › Supplementary_Figures/Figure_S3_regression_diagnostics.pdf]

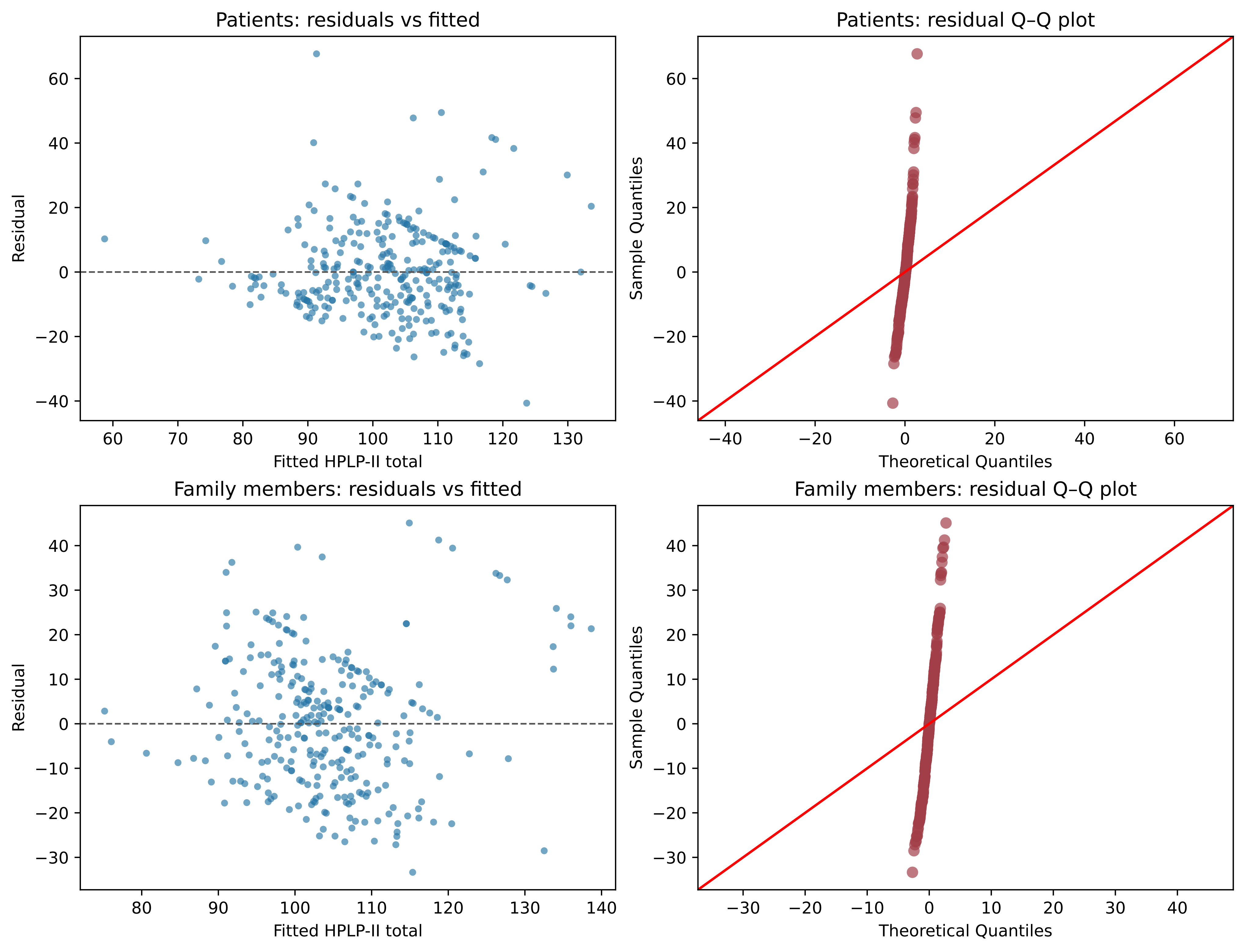
Patients: residuals vs fitted
Residual
Fitted HPLP-II total
Patients: residual Q–Q plot
Sample Quantiles
Theoretical Quantiles
Family members: residuals vs fitted
Residual
Fitted HPLP-II total
Family members: residual Q–Q plot
Sample Quantiles
Theoretical Quantiles
