# Supplementary material for: Health-Promoting Lifestyle and Psychosocial Correlates in Patients with NAFLD/MASLD and Family Members: A Comparative Cross-Sectional Study of Independent Samples
Source: Healthcare (Basel). 2026 Aug 24;14(17):2686. doi: 10.3390/healthcare14172686 (PMC13565544; doi:10.3390/healthcare14172686)
Supplement: Supplementary file 1 [file healthcare-14-02686-s001.zip › Supplementary_Materials.pdf]

Health-Promoting Lifestyle and Psychosocial Correlates in Patients with NAFLD/MASLD and Family Members: A Comparative Cross-Sectional Study of Independent Samples

Supplementary Materials

Table S1. Primary regression diagnostics

Sample	n	R^2	Adjusted R^2	Shapiro–Wilk W (p)	Breusch–Pagan LM (p)	RESET F (p)	Durbin–Watson	Maximum VIF
Patients	296	0.353	0.340	0.957 (<0.001)	10.461 (0.107)	1.170 (0.280)	1.881	1.52
Family members	290	0.290	0.275	0.986 (0.005)	41.076 (<0.001)	13.681 (<0.001)	1.654	1.56

VIF, variance-inflation factor. HC3 robust inference was emphasized because residual normality was rejected in both samples and heteroscedasticity was detected in the family-member model. RESET evidence of nonlinearity was also detected in that model.

Table S2. Expanded sensitivity-model coefficients

Predictor	Patients B (p), n=279	Family members B (p), n=289
Self-efficacy	0.546 (<0.001)	0.356 (<0.001)
Perceived social support	0.384 (<0.001)	0.501 (<0.001)
Sleep duration	1.385 (0.088)	0.929 (0.366)
Current smoking	-0.716 (0.825)	-4.408 (0.442)
Current alcohol use	-5.133 (0.100)	0.120 (0.983)
Disease duration	0.755 (0.010)	—

The expanded models included the six primary-model predictors plus sleep duration, current smoking, current alcohol use, and patient disease duration when available. Reported p values are conventional OLS sensitivity estimates; primary-model inference used HC3 covariance.

Table S3. Exploratory indirect-association and reverse-order decompositions

Sample	Order	n	a	b	Indirect B	Indirect β	Bootstrap 95% CI	Direct B	Total B
Patients	Support → self-efficacy → HPL	302	0.771	0.461	0.355	0.205	0.234 to 0.512	0.416	0.771
Patients	HPL → self-efficacy → support	302	0.506	0.206	0.104	0.181	0.057 to 0.162	0.151	0.255

Family members	Support → self-efficacy → HPL	293	0.558	0.292	0.163	0.092	0.071 to 0.287	0.466	0.629
Family members	HPL → self-efficacy → support	293	0.335	0.166	0.055	0.098	0.018 to 0.101	0.162	0.218

All decompositions adjusted for age and sex. Intervals used 5000 bias-corrected bootstrap resamples. These contemporaneous decompositions do not establish temporal order or causal mediation.

Table S4. Complete-case missingness assessment

**Sample**	**Variable**	**Included n**	**Included median**	**Excluded n**	**Excluded median**	**Mann–Whitney p**
Patients	HPLP-II total	296	101.0	9	102.0	0.342
Patients	Self-efficacy	296	71.0	9	82.0	0.370
Patients	Perceived social support	296	67.0	9	75.0	0.270
Patients	Autonomous motivation	296	48.0	9	48.0	0.568
Family members	HPLP-II total	290	104.0	9	102.0	0.832
Family members	Self-efficacy	290	74.0	9	69.0	0.685
Family members	Perceived social support	290	67.0	9	76.0	0.538
Family members	Autonomous motivation	290	48.0	9	52.0	0.725

Complete-case exclusion resulted from missing cleaned age or BMI. No main scale item was missing in a completed export. These comparisons cannot prove that data were missing at random.

Supplementary Figures

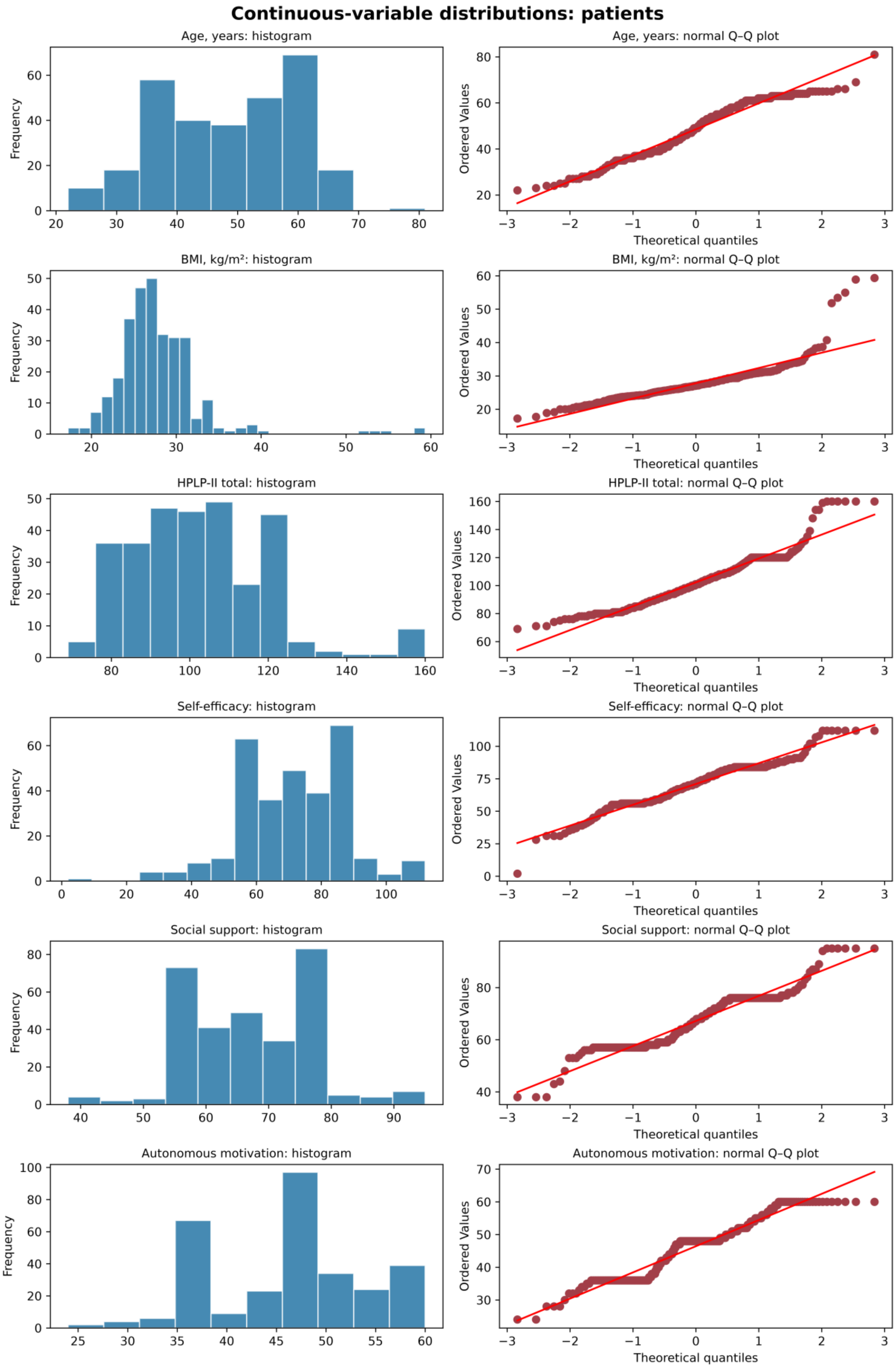

Figure S1. Histograms and normal Q–Q plots for continuous study variables in patients.

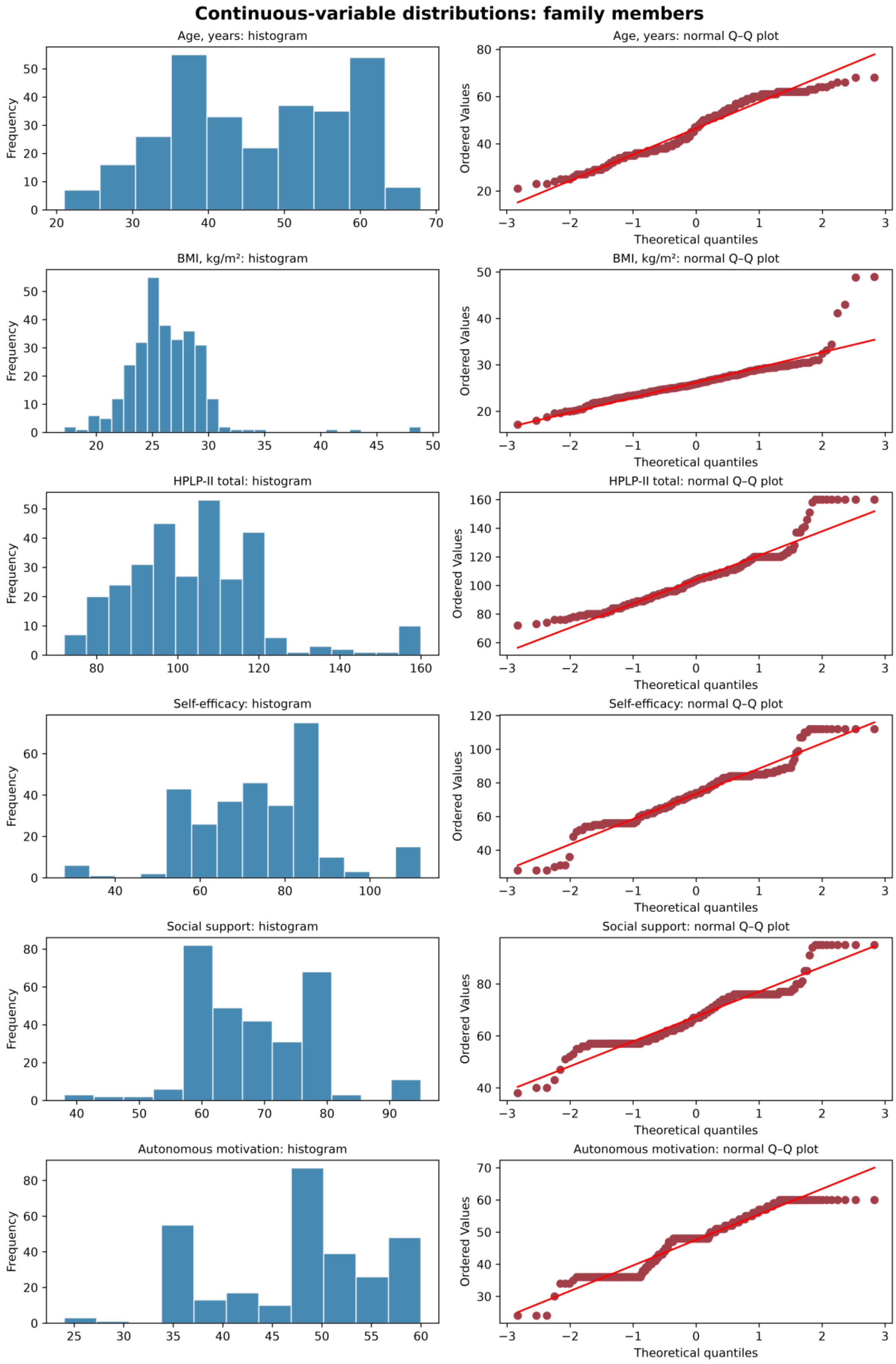

Figure S2. Histograms and normal Q–Q plots for continuous study variables in family members.

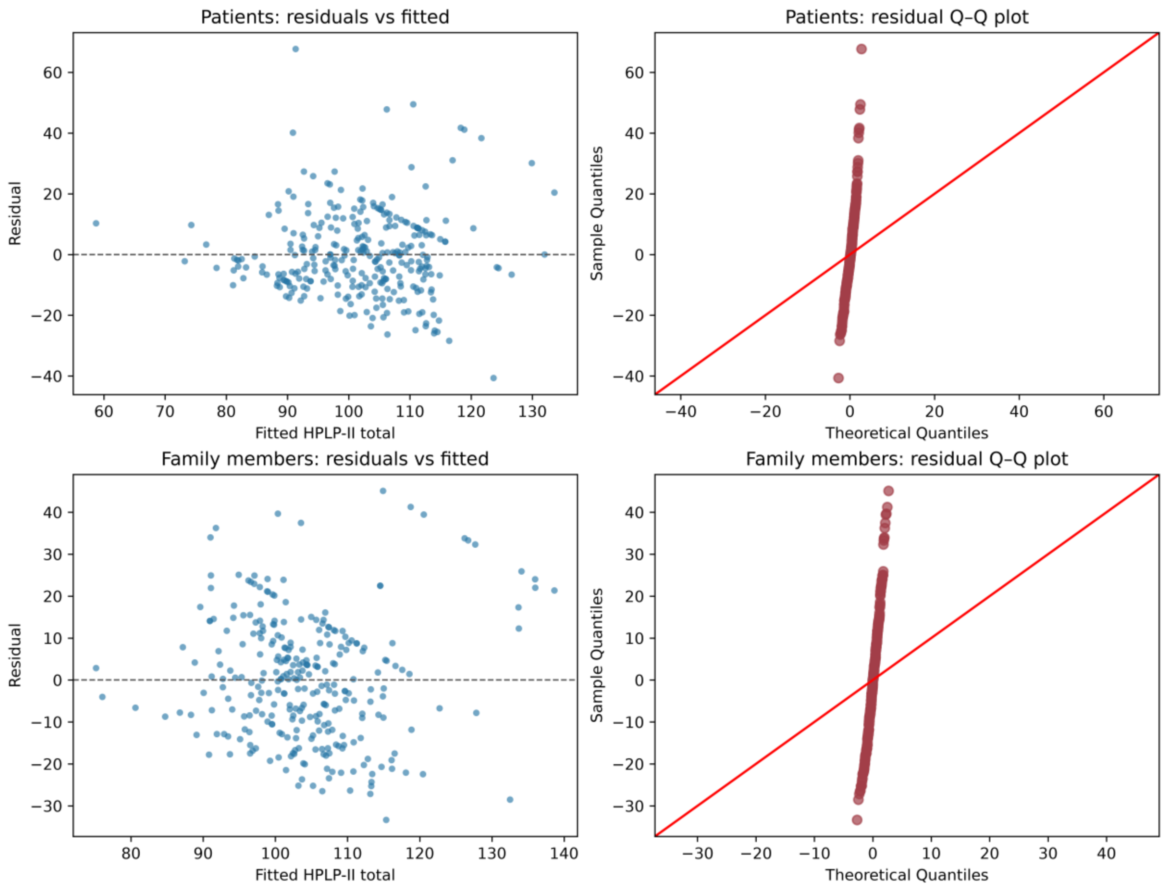

Figure S3. Residual-versus-fitted and residual normal Q–Q plots for the primary patient and family-member regressions.
